# Antiparasitic Activity of *Plumbago auriculata* Extracts and Its Naphthoquinone Plumbagin against *Trypanosoma cruzi*

**DOI:** 10.3390/pharmaceutics15051535

**Published:** 2023-05-19

**Authors:** Raiza Brandão Peres, Marcos Meuser Batista, Ana Luíza Rangel Bérenger, Flávia da Cunha Camillo, Maria Raquel Figueiredo, Maria de Nazaré Correia Soeiro

**Affiliations:** 1 Laboratório de Biologia Celular, Instituto Oswaldo Cruz, Fundação Oswaldo Cruz (FIOCRUZ), Rio de Janeiro 210360-040, Brazil; raizabperes@gmail.com (R.B.P.); meusermb@ioc.fiocruz.br (M.M.B.); 2 Laboratório de Tecnologia para Biodiversidade em Saúde-TecBio/LDFito, Instituto de Tecnologia em Fármacos (Farmanguinhos), Fundação Oswaldo Cruz (FIOCRUZ), Rio de Janeiro 21041-250, Brazil; ana.berenger@fiocruz.br (A.L.R.B.); flavia.camillo@fiocruz.br (F.d.C.C.); maria.figueiredo@fiocruz.br (M.R.F.)

**Keywords:** Chagas disease, natural products, *Plumbago auriculata*, Plumbagin, naphthoquinone, drug combination, in silico ADMET

## Abstract

Chagas disease (CD) caused by the protozoan *Trypanosoma cruzi* affects more than six million people worldwide. Treatment is restricted to benznidazole (Bz) and nifurtimox (Nf) that display low activity in the later chronic stage besides triggering toxic events that result in treatment abandonment. Therefore, new therapeutic options are necessary. In this scenario, natural products emerge as promising alternatives to treat CD. In the family Plumbaginaceae, *Plumbago* sp. exhibits a broad spectrum of biological and pharmacological activities. Thus, our main objective was to evaluate, in vitro and in silico, the biological effect of crude extracts of root and of aerial parts of *P. auriculata*, as well as its naphthoquinone Plumbagin (Pb) against *T. cruzi*. The phenotypic assays revealed potent activity of the root extract against different forms (trypomastigote and intracellular forms) and strains (Y and Tulahuen), with a compound concentration that reduced 50% of the number of the parasite (EC_50_) values ranging from 1.9 to 3.9 µg/mL. In silico analysis showed that Pb is predicted to have good oral absorption and permeability in Caco2 cells, besides excellent probability of absorption by human intestinal cells, without toxic or mutagenic potential effects, not being predicted as a substrate or inhibitor of P-glycoprotein. Pb was as potent as Bz against intracellular forms and displayed a superior trypanosomicidal effect (about 10-fold) in bloodstream forms (EC_50_ = 0.8 µM) as compared to the reference drug (8.5 µM). The cellular targets of Pb on *T. cruzi* were evaluated using electron microscopy assays and the findings on bloodstream trypomastigotes showed several cellular insults related to the autophagic process. Regarding toxicity in mammalian cells, the root extracts and the naphthoquinone present a moderate toxic profile on fibroblasts and cardiac cell lines. Then, aiming to reduce host toxicity, the root extract and Pb were tested in combination with Bz, and the data showed additive profiles with the sum of the fractional inhibitory concentration indexes (ΣFICIs) being 1.45 and 0.87, respectively. Thus, our work reveals the promising antiparasitic activity of *Plumbago auriculata* crude extracts and its purified naphthoquinone Plumbagin against different forms and strains of *Trypanosoma cruzi* in vitro.

## 1. Introduction

Existing in the America continent for more than 9000 years, Chagas disease (CD) or American Trypanosomiasis, first reported in 1909 by the sanitary doctor Carlos Chagas, is caused by the intracellular protozoan *Trypanosoma cruzi* [1,2,3]. Currently, CD is endemic in 21 countries in Latin America, with more than 6 million infected individuals, approximately 12,000 deaths per year and an estimated 75 million people at risk [4]. *T. cruzi* is transmitted by different ways including the vector route, which is considered to be the classic mode as seen via the presence of the parasite in the feces/urine of the triatomine insect eliminated after/during its blood meal [3,5]. Other transmission routes include blood transfusion, organ transplants, vertical transmission (from mother to child, during pregnancy or childbirth) or orally via food contamination, among others of lesser relevance such as laboratory accidents [3,5]. Oral transmission has become an important concern in recent decades, mainly in the Amazon region, accounting for more than 70% of new acute cases [6,7]. Additionally, increased population mobility, due to economic and/or political reasons, has resulted in CD globalization, reaching non-endemic territories such as North America, Europe and Asia [8].

CD has two clinical consecutive phases: acute and chronic. Lasting up to eight weeks, the acute phase is characterized by patent parasitemia and an asymptomatic/oligosymptomatic profile. Due to a competent host immune response, the parasitic proliferation is controlled, but not extinguished, and then the infected individual progresses to the chronic phase of the disease [5,9]. In this later stage, undetectable and intermittent levels of parasites in the blood are observed, characterizing subpatent parasitemia of the chronic phase [10,11]. In most cases, the absence of clinical signs and symptoms are observed; however, 30–40% of the carriers will develop, years or even decades after infection, cardiac and/or digestive alterations [12].

Regarding treatment, the available therapeutic arsenal is restricted to two nitroderivatives introduced in clinical medicine more than 50 years ago: nitrofuran nifurtimox—Nf, and nitroimidazole benznidazole—Bz [13,14]. Administered orally, their nitro group is metabolically reduced by the parasite’s nitroreductase type I enzymes, resulting in highly toxic metabolites [15,16]. Although effective in the acute phase, both drugs have limitations such as low efficacy during the chronic phase of the disease and serious toxicity, in addition to the existence of strains naturally resistant to nitroderivatives [3,7,17]. To deal with these limitations, the search for new drugs is extremely important and necessary.

Natural products have been exploited for therapeutic purposes since ancient times to alleviate and cure ailments, and products obtained and/or derived from plant sources have been used to treat several diseases [18]. Diverse natural plant products have been reported to have antitrypanosomal activity including triterpenes, diterpenes, sesquiterpene, alkaloids and flavonoids [19,20,21,22].

*Plumbago auriculata* Lam. (Plumbaginaceae) is a perennial shrub, native to South Africa, ranging from the Southern Cape to the Eastern Cape and into KwaZulu-Natal. It has been introduced in tropical and subtropical countries of Europe, Asia and the Americas. In Brazil, the species is very well adapted and commonly found in gardens due to its ability to flower throughout the year [23,24]. Several *Plumbago* species are used in folk medicine to treat wounds, warts, fractures and headaches, presenting anti-inflammatory, antimicrobial, anticancer and hepatoprotective activities [23,24,25,26].

The chemical profile of the species is characterized by the presence of naphthoquinones, flavonoids, terpenoids and steroids, with the first two being considered to be chemosystemic markers of the Plumbaginaceae family [27]. Phytochemical studies of *P. auriculata* have resulted in the isolation of sitosterol, stigmasterol, 3-*O*-glycosylsitosterol, palmitic acid, plumbagic acid, epi-isoshinanolone, luteolin, 5-methoxyluteolin and plumbagin. The bioactive naphthoquinone plumbagin (C_11_H_8_O_3_) has an antitumor effect [28,29,30] and is active against multidrug-resistant clinical isolates of bacteria [31,32], besides presenting molluscicidal and antischistosomiasis [33], antimalarial [34] and antileishmanial [35] properties.

Thus, due to previous reports of the biological properties of *Plumbago auriculata* and its naphthoquinone Plumbagin, our aim was to investigate their potential antiparasitic and pharmacological effects using in vitro and in silico approaches against different forms and strains of *Trypanosoma cruzi*.

## 2. Materials and Methods

### 2.1. Plant Material

Aerial parts and roots of *Plumbago auriculata* were collected at the Fundação Oswaldo Cruz campus, Manguinhos, Rio de Janeiro, Brazil. The georeferencing of the species was S22°52′35.6″ S 43°15′04.6″ W. A witness specimen was deposited at the Herbário Jardim Botânico (RB) in Rio de Janeiro under number 193592. This plant is registered with the National Genetic Heritage Management System-SisGen under number AB5D582, in compliance with the provisions of Law No. 13.123/2015.

### 2.2. Plant Extraction

Flowers, leaves, branches and roots were separated and dried at 40 °C and reduced to small fragments (5.0 mm particle diameter). The plant material, respectively, 12.0 g, 94.0 g, 123.0 g and 141.0 g, was submitted separately for dynamic maceration with ethanol at room temperature in a proportion of 1/10 plant/solvent for 72 h. Subsequently, all extracts were filtered and concentrated under reduced pressure, obtaining yields of 2.6%, 18.0%, 13.0% and 8.0% for crude ethanol extracts of each respective part of the species. These extracts were prepared at the laboratory of natural products, TecBio/LDFito, Farmanguinhos/FIOCRUZ.

### 2.3. Plumbagin Isolation

Plumbagin, mainly present in the roots, is responsible for some reported biological activities of the *Plumbago* species. Due to these properties, only the root extract of the plant was used in the separation process.

The ethanol extract of the root (11.3 g) was suspended in water: ethanol (9:1, *v*/*v*) and partitioned using chloroform. After drying under reduced pressure, the chloroform fraction (5.0 g) was submitted to fractionation via column chromatography using Merck silica gel 60 (0.063–0.200 mm) in the stationary phase, eluted with *n*-hexane-ethyl acetate (2–50%), ethyl acetate 100% and methanol 100%. The separation led to us obtaining 183 fractions. The fraction eluted with the *n*-hexane/ 2% ethyl acetate solvent system yielded 0.75 g of a crystalline solid in the form of yellow needles. These crystals were analyzed and identified as being Plumbagin, using ^1^H and ^13^C nuclear magnetic resonance (NMR) spectrometric methods (see Supplementary Materials).

### 2.4. NMR Spectroscopy

One-dimensional (^1^H, ^13^C, DEPT-135) and two-dimensional (COSY, HSQC, HMBC) NMR technique spectra were acquired using a Bruker (Rheinstetten, Baden-Württemberg, Germany) equipment model DRX 400 at 400.13 and 100.61 MHz, respectively, and a Bruker equipment model Advance 500 at 500.13 and 125.77 MHz, respectively. The solvent used on all of the NMR experiments was CDCl_3_ and the internal reference was TMS.

### 2.5. Compounds

Bz was used as the reference drug and was purchased from Laboratório Farmacêutico do Estado de Pernambuco (Brazil). The plant samples were prepared as stock solutions in dimethylsulfoxide (DMSO; Sigma-Aldrich, St. Louis, MO, USA) and used as the final working concentrations, never exceeding 0.6%, meaning no toxic effect for the mammalian cells or parasites [36].

### 2.6. Parasites

Bloodstream trypomastigote forms of *T. cruzi* (Y strain, Discrete Typing Units (DTU) II) were obtained via cardiac puncture of infected Swiss male mice [37]. The trypomastigote forms of the Tulahuen strain (DTU VI) expressing the *E. coli β*-galactosidase gene were collected from the supernatant of mouse fibroblast cell lines (L929) that were previously infected (host/parasite cell ratio 10:1) [38]. For both strains, purified parasites were added to RPMI 1640 medium supplemented with 5% fetal bovine serum (FBS) to perform assays at 37 °C in 5% CO_2_. All animal studies were carried out in strict accordance with the guidelines established by the FIOCRUZ Committee of Ethics for the Use of Animals (CEUA L038-2017).

### 2.7. Mammalian Cells

Primary cultures of cardiac cells (CCs) were obtained from the embryonic hearts of Swiss Webster mice (18 days of gestation); after purification, cardiac cells were seeded (0.5 × 10^5^ cells/well) into 96-well microplates previously coated with 0.01% gelatin [27].

L929 cell lines were routinely maintained via weekly dissociation with 0.01% trypsin solution. Cells were seeded (4 × 10³ cells/well) in 96-well microplates and sustained at 37 °C in RPMI 1640 medium (Sigma-Aldrich, MO, USA) supplemented with 10% FBS, 1% L-glutamine and 1% penicillin [39]. Cell cultures were maintained at 37 °C in 5% CO_2_.

### 2.8. Cytotoxicity Assay

CC and L929 cells were incubated for 24 and 96 h with increasing concentrations up to 400 µg/mL or 400 µM of the plant samples serially diluted in Eagle’s medium or RPMI-1640 medium, respectively, and supplemented with 1% L-glutamine and 10% FBS [36]. Aspects of cell morphology were evaluated under a light microscope, as well as contractability (for CC), cellular density and cytoplasmic vacuolization. Cellular viability was further assessed using PrestoBlue (for CC assay) and AlamarBlue (for L929 assay) assays with fluorescence and absorbance readouts (560–590 and 570–600 nm, respectively) in a spectrophotometer [36]. Cellular viability rates were then determined using LC_50_ values that corresponded to the concentration of the compound capable of reducing the viability of the cell population by 50% [36].

### 2.9. Transmission Electron Microscopy (TEM) Analysis and Scanning Electron Microscopy (SEM)

Bloodstream trypomastigotes (BTs) were used for TEM and SEM analyses. The parasites were exposed for 2 and 24 h at 37 °C with Plumbagin (at the concentration corresponding to its EC_50_ for 24 h), rinsed with phosphate buffered saline solution (PBS) and fixed for 60 min at 4 °C with glutaraldehyde at 2.5% diluted in 0.1 M cacodylate buffer, pH 7.2. For TEM analysis, the parasites were washed 3 times with 0.1 M sodium cacodylate buffer and post-fixed for 15 min with 1% osmium tetroxide (OsO4), 0.8% potassium ferricyanide and 5 mM CaCl_2_ in 0.1 M cacodylate buffer, pH 7.2. Then, samples were washed with the same buffer, dehydrated in an ascending acetone series and embedded in PolyBed 812 resin, as described [40]. Ultrathin sections were stained with uranyl acetate and lead citrate and examined under a transmission electron microscope at Platform Rudolf Barth in Instituto Oswaldo Cruz, FIOCRUZ. For SEM analysis, the fixed parasites were adhered to glass coverslips coated with 0.1% poly-L-lysine, and post-fixed as described above. Then, samples were dehydrated in a crescent ethanol series (30–100%), dried using the critical point method with CO_2_, mounted on aluminum stubs coated with a 20 nm gold layer and examined using a Jeol JSM6390LV scanning electron microscope (Tokyo, Japan) at Platform Rudolf Barth in Instituto Oswaldo Cruz, FIOCRUZ [41,42].

### 2.10. Trypanocidal Activity

Against the intracellular forms, the antiparasitic effect of the plant samples was evaluated after 48 and 96 h of compound (up to 100 µg/mL or 100 µM) incubation using L929 cell lines (infected with tissue-culture-derived trypomastigotes of Tulahuen *β*-gal strain) and CC (infected with bloodstream trypomastigotes of Y strain), respectively, always using a parasite/host cell ratio of 10:1 [39,43]. The cultures were incubated at 37 °C/5% CO_2_. Bz and DMSO (solvent used for the compounds) were run in parallel as positive and negative controls, respectively. The activity of the compounds was expressed by the EC_50_ and EC_90_ values, which represented the concentrations capable of inducing a 50% and 90% loss of viability in the parasites, respectively.

The antiparasitic activity of the plant samples against BT (Y strain) was also investigated. Briefly, 100 μL of a BT suspension (in RPMI medium + 5% FBS) containing 10^7^ parasites/mL was added to the same volume of each compound diluted in RPMI + 5% FBS at twice the desired final concentration (serially diluted 1:2, up to 100 µg/mL or 100 µM). After 2 and 24 h at 37 °C, the number of live parasites was determined via light microscope quantification using a Neubauer chamber. Untreated control samples were carried out with parasites kept under the same conditions in the absence of the compounds. Bz was run in parallel. The activity of the compounds was expressed by the EC_50_ and EC_90_ values (compound concentrations that reduced by 50 and 90% the number of the parasites) [44].

### 2.11. Determination of Drug Interactions against T. cruzi

In vitro drug interactions were evaluated based on the EC_50_ determined via intracellular forms of the parasite (Tulahuen *β*-gal strain). The reference drug (Bz) was used and combined with the root extract and with Pb under fixed proportions of 5:0, 4:1, 3:2, 2:3, 1:4 and 0:5, as reported [45]. The fractional inhibitory concentrations index (FICI) and the sum of FICI (∑FICI) were determined, and the isobologram was constructed to classify the nature of the interaction between the tested compounds and classify the nature of each interaction, ∑FICs ≤ 0.5 = synergism; 0.5 < ∑FICs ≤ 4.0 = additive (no interaction); ∑FICs > 4.0 = antagonism [46].

### 2.12. In Silico

Absorption, distribution, metabolism, excretion and toxicity (ADMET) and Lipinski’s rule of five properties of Bz and Pb were assessed as perspectives in an in silico analysis for estimating drug-likeness and oral bioavailability using the predicting small-molecule pharmacokinetic and toxicity properties (pKCSM) approach, which uses graph-based signatures to develop predictive ADMET [47].

### 2.13. Statistical Analysis

The analyses were performed using GraphPad Prism v9.0 (GraphPad Software, San Diego, CA, USA). Statistical analysis was performed using a one-way ANOVA test with the significance level set at *p* ≤ 0.05.

### 2.14. Ethics

All animal studies were carried out in strict accordance with the guidelines established by the FIOCRUZ Committee of Ethics for the Use of Animals (CEUA L038-2017).

## 3. Results

### 3.1. Crude Extract Activity

The first set of the in vitro phenotypic screening evaluated the activity of crude extracts from the root as well as the aerial parts (branches, leaves and flowers) of *Plumbago auriculata* against the intracellular forms of *T. cruzi*, comparing it to the effect of Bz. The intracellular forms of the parasite (Tulahuen strain, 48 h of L929 infection) were exposed for 96 h to increasing concentrations of the extracts. As depicted in Table 1, the root extracts showed potent antiparasitic activity, revealing an EC_50_ of 3.6 μg/mL. On the other hand, the aerial parts of the plant (leaves, flowers and branches) presented EC_50_ > 100 μg/mL (Table 1). The evaluation of the cytotoxicity on mammalian cells was carried out in parallel using mouse fibroblasts (L929 cell line). Cultures of uninfected L929 cells were exposed or not to the crude extracts and Bz, and after the 96 h of incubation, the cell viability was measured using a colorimetric assay to determine the LC_50_ values. Our finding corroborated the literature data demonstrating the lack of in vitro toxicity of Bz up to 100 µg/mL [25,27] (Table 1). The extracts obtained from the aerial parts (branches, leaves and flowers) did not show toxic profiles against L929 cell lines, but the root extract presented an LC_50_ value of 29.6 µg/mL, resulting in a selectivity index = 8.2 (Table 1).

Next, the extracts were assessed against the other relevant parasite forms for mammalian infection and bloodstream trypomastigotes (BTs). BTs were incubated for 2 and 24 h with increasing concentrations of the samples, with only culture medium or with the reference compound (Bz), used as negative and positive controls, respectively. The number of live/motile parasites was then quantified via light microscopy using Neubauer chambers for the determination of the EC_50_.

Surprisingly, after a short time of incubation (2 h), the root extract of *Plumbago auriculata* reached an EC_50_ of 30.1 µg/mL, while Bz and the extracts of the aerial samples were inactive up to the maximum tested concentration (Table 1). After 24 h of incubation, Bz showed EC_50_ and EC_90_ values of 2.96 and 5.9 µg/mL, respectively (Table 1). After 24 h of incubation, the extracts obtained from the leaves and flowers were inactive against the parasite (Table 1). The extracts obtained from the branches showed moderate antiparasitic activity, revealing an EC_50_ of 41.3 µg/mL. As observed against intracellular forms, the root extract of *Plumbago auriculata* was very active, displaying EC_50_ and EC_90_ values of 1.9 and 5.6 µg/mL, respectively (Table 1). All extracts did not present a cardiotoxic profile when assayed for 24 h on CC up to 100 μg/mL, and then the root extract presented a promising selectivity index > 53 (Table 1).

### 3.2. Plumbagin Isolation

**Figure 1:**
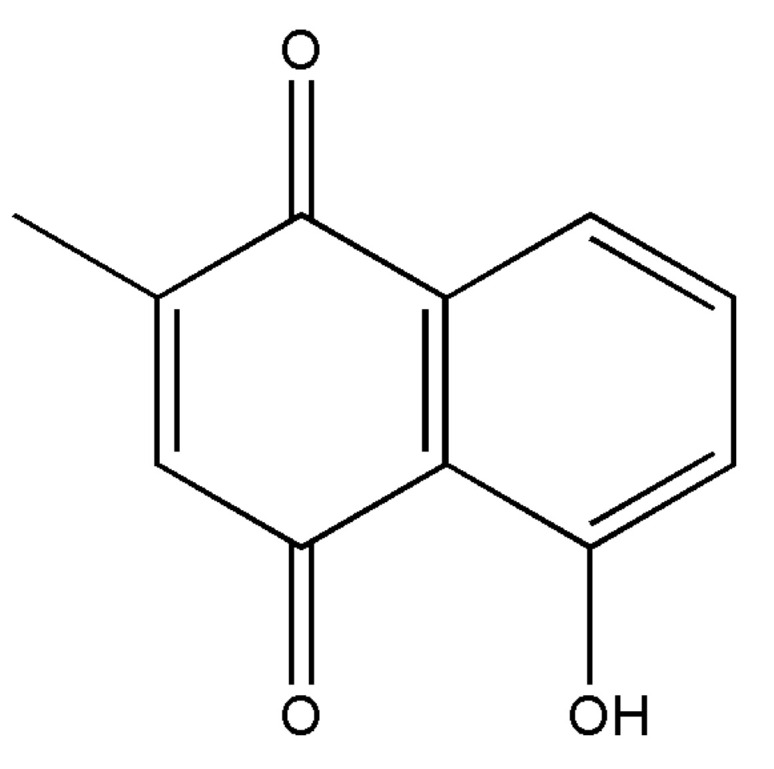
Chemical structure of 5-hydroxy-2-methyl-1,4-naphthoquinone (Plumbagin).

Due to the in vitro antiparasitic findings achieved with the root extract of *Plumbago* sp. [48], chromatographic purification was performed next and 5-hydroxy-2-methyl-1,4-naphthoquinone (Plumbagin-Pb) was isolated and tested (Figure 1). This naphthoquinone was evaluated using ^1^H and ^13^C-NMR spectrometric methods and the data (Figures S3 and S4) were similar to those reported in the literature [49,50].

Then, the pharmacological profile of Pb was investigated via in silico analysis using the pKCSM tool. The results showed that, similarly to Bz, Pb does not violate Lipinski’s rule of five, with a prediction of good oral absorption (Table 2).

Plumbagin showed a good probability of permeability in Caco2 cells, with values above the hypothetical threshold of 0.9 log cm/s, besides an excellent probability (>90%) of absorption by the human intestinal cells (Table 3). This naphthoquinone is neither a substrate nor an inhibitor of P-glycoprotein (P-gp), unlike Bz which is a substrate. Both of them (Bz and Pb) are not inhibitors of most cytochrome P450 isoforms, and plumbagin was predicted not to be an human-ERG (hERG) blocker and non-mutagenic and non-hepatotoxic activity, unlike Bz that has both mutagenic and hepatotoxicity profiles (Table 3).

The activity of Pb was assayed. The results depicted in Table 4 demonstrate its excellent in vitro anti-*T. cruzi* effect, revealing the EC_50_ value against the intracellular forms to be in the same range as the reference drug (2.4 and 3.2 µM for Bz and Pb, respectively). Regarding toxicity against mammalian cells, light microscopy inspection did not reveal toxic events on morphology and cellular density of L929 and CC cultures, for both Bz and Pb, after 2 h of incubation. When L929 cell lines were incubated for 24 h, while Bz showed LC_50_ values higher than 200 µM, Pb gave an LC_50_ value of 12 µM, being about two-fold more toxic than the crude root extract (Table 1 and Table 4).

Next, Pb was tested on bloodstream trypomastigotes. The data revealed the potent trypanocidal action against this parasite form even after short periods of compound exposure (2 h), with EC_50_ and EC_90_ values of 2.6 and 6.15 μM, respectively, while Bz was >50 μM (Table 4) [51]. With longer exposure times (24 h of incubation), plumbagin sustained its high antiparasitic activity on the submicromolar scale, revealing EC_50_ and EC_90_ values equal to 0.8 and 1.7 μM, respectively, being at least ten-fold more potent than the reference drug (EC_50_ and EC_90_ values of 8.5 and 40.7 µM, respectively). The incubation of uninfected CC for 24 h with Pb showed a fairly toxic profile, with an LC_50_ value = 1.35 μM, while Bz did not exert toxic events up to the maximum tested concentration (Table 4).

**Figure 2:**
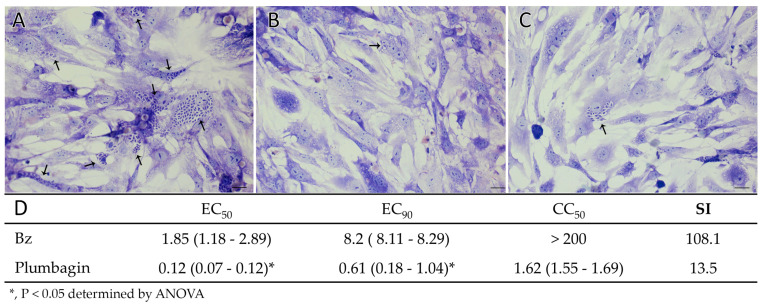
Light microscopy images of CC cultures infected with *T. cruzi* (Y strain) (**A**–**C**) or untreated (**A**), or exposed to 0.37 µM plumbagin (**B**) and 0.12 µM plumbagin (**C**). Bars = 20 μm. Phenotypic activity (**D**) (EC_50_ and EC_90_ μM) of plumbagin and benznidazole (Bz) against intracellular forms (Y strain) of *T. cruzi* and cytotoxicity (LC_50_, μM) upon primary cardiac cell (CC), with their respective selectivity indexes (SIs). Values in parentheses are standard errors of the mean with 95% confidence intervals. Arrows: intracellular parasites.

Furthermore, the effect of Pb on *T. cruzi*-infected CC was investigated. As shown in Figure 2, plumbagin is highly active against intracellular forms within CC at concentrations that do not induce toxic alterations in the cellular morphology and density of the host cells (Figure 2), reaching EC_50_ value = 0.12 μM, being about fifteen-fold more active than Bz (Figure 2).

**Figure 3:**
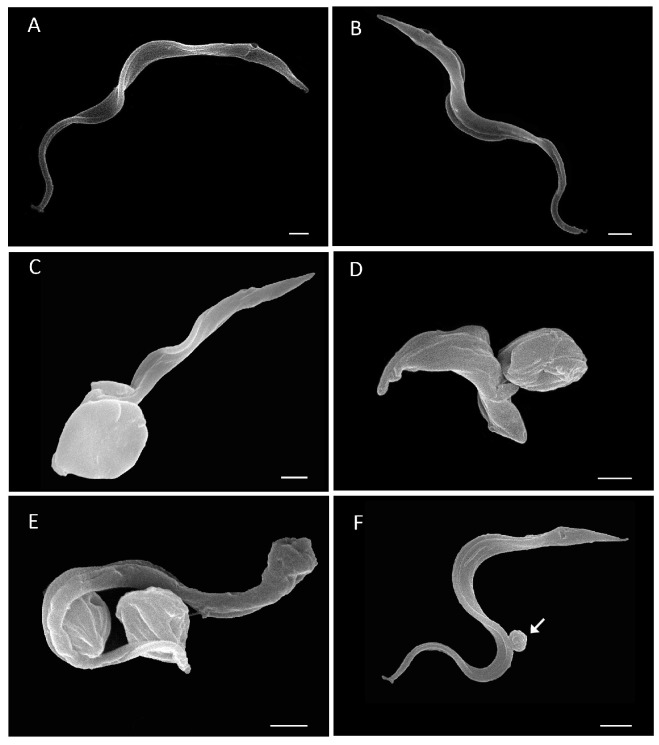
Scanning electron microscopy (SEM) examination of *T. cruzi* bloodstream trypomastigotes (Y strain) treated for 2 h (**C**) and 24 h (**D**–**F**) with Plumbagin at 0.8 μM (corresponding to EC_50_/24 h). Untreated parasites ((**A**) 2 h, and (**B**) 24 h) showing typical morphology. Bloodstream forms exposed to Plumbagin displayed several morphological alterations including body retraction (**C**,**E**), a twist of the parasite body (**D**) and profuse shedding events ((**F**) white arrows). Bars = 1 μm.

To explore the cellular insults in *T. cruzi* triggered by plumbagin, ultrastructural studies were performed on bloodstream trypomastigotes incubated for 2 h and 24 h, with the EC_50_ corresponding to 24 h (0.8 μM). Scanning electron microscopy (SEM) showed several morphological alterations after 2 h (Figure 3C) and 24 h (Figure 3D–F) of incubation with Plumbagin, including body retraction (Figure 3C,E), a twist of the parasite body (Figure 3D) and profuse shedding events (Figure 3F).

**Figure 4:**
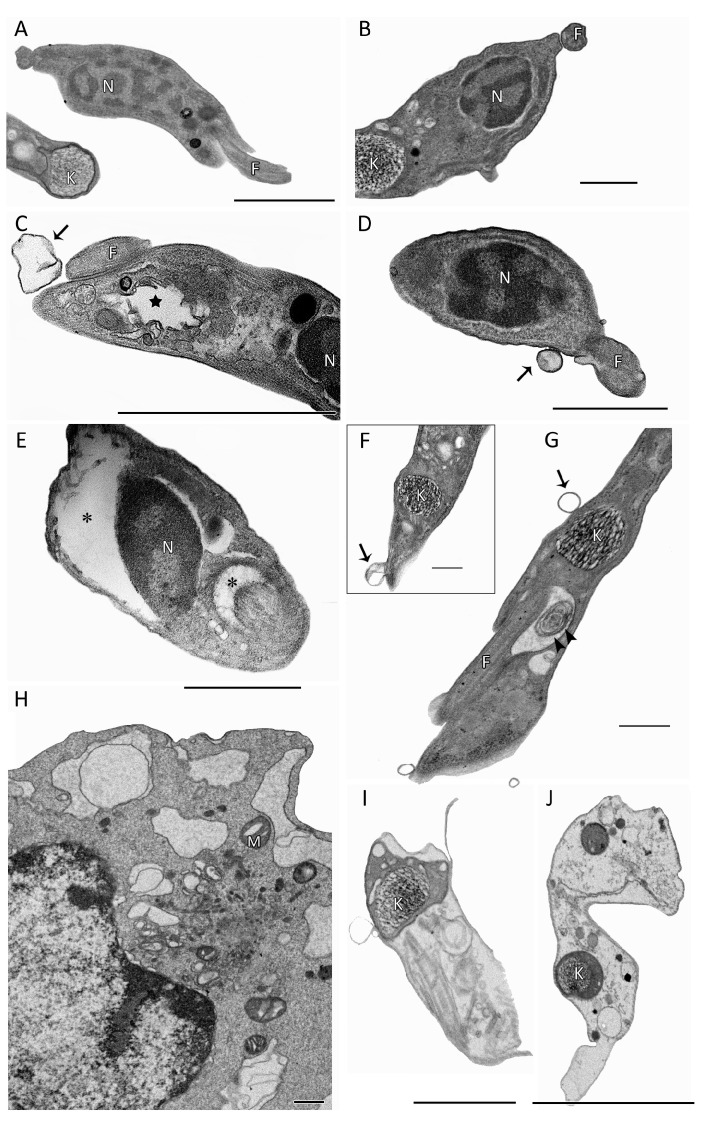
Ultrastructural analysis: transmission electron microscopy (TEM) of bloodstream trypomastigotes (Y strain) treated for 2 h with Plumbagin at 0.8 μM (corresponding to EC_50_/24 h). (**A**,**B**) Untreated parasites showing typical morphology. (**C**–**I**) Bloodstream forms exposed to Plumbagin displayed several morphological alterations including profuse shedding events ((**C**,**D**,**F**,**G**) black arrows), a swollen nuclear envelope ((**E**) asterisk) and flagellar pockets ((**E**) asterisk), insults in Golgi (**C**, black star), the presence of concentric membranes and myelin figures ((**G**) arrowhead), a high number of intracellular dilated vesicles (**H**–**J**) and numerous ghost parasites with retained KDN and microtubules (**I**,**J**). N, nuclei; F, flagellum; K, kinetoplast; M, mitochondria. Bars = 1 μm.

As indicated via TEM, the main cellular alterations induced by Pb exposure of bloodstream forms included profuse shedding events (Figure 4C,D,F,G), a swollen nuclear envelope (E, asterisk), enlarged flagellar pockets (Figure 4E), insults in Golgi (Figure 4C), the presence of concentric membranes and myelin figures (Figure 4G), a high number of intracellular dilated vesicles (Figure 4H–J) and numerous ghost parasites with retained KDN and microtubules (Figure 4I,J), an apparent sign of autophagy.

**Figure 5:**
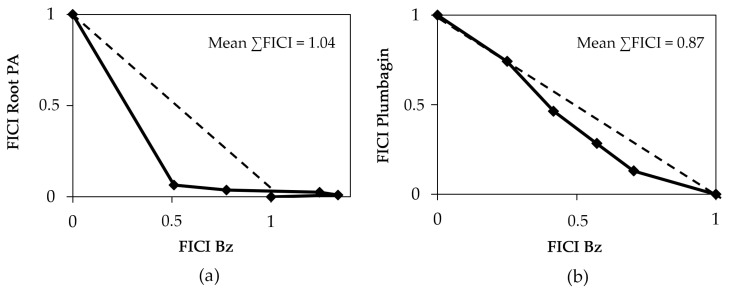
Phenotypic activity (ΣFIC_50_) of the combination composed of the root extracts of Plumbago auriculata (PA) plus benznidazole (Bz) (**a**) and of Plumbagin plus benznidazole (Bz) (**b**) against intracellular forms (Tulahuen strain) of *T. cruzi* after 96 h of treatment.

The excellent activity of Pb on *T. cruzi* but its fairly toxic profile against mammalian host cells prompted us to perform drug combination assays in an attempt to reduce toxicity whilst promoting antiparasitic activity. The toxicity of the combinations was inspected via light microscopy, assessing the cellular density and general morphology that were not affected. Then, drug interaction assays were conducted using the crude root extract of Plumbago auriculata and Pb with Bz (Figure 5). The mean fractional inhibitory concentration indexes (FICIs) showed that both combinations were classified as additive with ∑FICI = 1.04 and 0.87 for root extract + Bz and Pb + Bz, respectively (Figure 5). The combination demonstrated all of the ∑FICI for each ratio, and none of them were synergistic.

## 4. Discussion

Natural products have been used since ancient times as folk medicine and represent a wide and continuous source of new chemical entities/scaffolds that can be exploited for therapeutic purposes [52]. The ingestion of infusions of herbs and leaves may have been one of the first uses of natural products [18].

The biological and pharmacological properties of extracts and fractions of *Plumbago* sp. have been reported in the literature, including hydroxynaphthoquinone Plumbagin, one of the main active constituents of plants of this species [18,19].

In fact, plumbagin, mainly present in the roots, is responsible for some of the reported biological activities of the *Plumbago* species [28]. Due to these properties, the activity of *Plumbago auriculata* was presently investigated on *T. cruzi* using different approaches in vitro.

The initial screenings evaluated the effect of the crude extracts (root, branches, leaves and flowers) of *P. auriculata* and revealed that the root extract is active against both parasite forms present in mammalian cells (intracellular and trypomastigotes) from distinct strains and DTUs (Tulahuen and Y strains belonging to DTUs VI and II, respectively). Our findings corroborate the literature data that describe a greater distribution of naphthoquinone in the roots of the *Plumbago* species [25,29].

The significant root extract activity is suggested to be due to the expressive presence of Plumbagin in this part of the plant. Future studies are needed to determine the amount of this naphthoquinone in other parts of *P. auriculata*.

Plumbagin (5-hydroxy-2-methyl-1,4-naphthoquinone) is a plant naphthoquinone obtained mainly from three families, including Plumbaginaceae, Droseraceae and Ebenaceae [53]. The potential therapeutic benefits of Plumbagin on different chronic conditions have been suggested in vitro and in vivo, presenting anticancer, anti-inflammatory, antioxidant and antimicrobial effects, as well as being suggested to control diabetes and cardiovascular diseases [53,54].

Presently, the chromatographic separation of roots of *P. auriculata* has resulted in the isolation and purification of Plumbagin. Then, as computer models can be used to predict pharmacological properties, in silico studies of Pb were performed next following the recommendation of the regulatory agencies such as the Food and Drug Administration (FDA) related to the early identification of structural alerts related to toxic, mutagenic and genotoxic profiles [55].

In silico analysis revealed that Pb is neither a substrate nor an inhibitor of P-glycoproteins. This is an important prediction considering the role of P-glycoprotein (P-gp) in drug absorption and disposition, and special attention should be paid to the substrate and inhibitory properties of new compounds.

Our results also demonstrated that plumbagin has neither an inhibition nor a substrate profile with the main enzymes of the cytochrome P540 family. Cytochrome P450 is the main metabolic enzyme to be considered in the process of drug metabolism, which can oxidize xenobiotics to facilitate their elimination [56]. The inhibition of these enzymes suggests the potential for the occurrence of toxicity and possible interference with the pharmacokinetics of co-administered compounds.

Plumbagin is additionally predicted to be a non-human-ERG (hERG) blocker, and to show non-mutagenic and non-hepatotoxic activity, unlike Bz. The cardiac potassium channel (K^+^) is encoded by the human ether-à-go-go-related gene (hERG). Due to its notorious ligand promiscuity, this ion channel has proven to be an important issue in the early drug discovery process [57].

Our present findings likewise corroborate the data presented by Mishra et al. [58] that reported the potent in vitro anti-*Leishmania* activity of naphthoquinones from the roots of *Plumbago zeylanica*. Our findings confirmed that the plumbagin purified from the roots of *Plumbago auriculata* is a potent antiparasitic agent, displaying high anti-*T. cruzi* activity in the same range as Bz (or even more active) against both strains (Tulahuen and Y strain, respectively) and forms of the protozoan. However, Pb showed a toxic profile upon cell lineages (L929) and cardiac cell cultures, requiring additional approaches (such as hit optimization) to sustain the antiparasitic activity and reduce the host cell toxicity.

In an attempt to reduce cytotoxicity, the combination of drugs appears to be a relevant therapeutic strategy [59]. Presently, we assayed combinations composed of “the reference drug + the root extract” and “the reference drug + Pb”. Our findings demonstrated that in both combinations, the interactions were classified as being additive (mean ΣFIC between >0.5 and ≤4.0), proving another promising result related to these natural products aiming to mitigate their toxicity and promote their efficacy [3].

Finally, ultrastructural analysis was performed to evaluate the possible cellular targets of Pb in bloodstream trypomastigotes. SEM and TEM revealed important morphological changes with a phenotype of an autophagic process that corroborated the studies of Menna-Barreto et al. [60] and Salomão et al. [61]. The authors reported autophagy as being the main mechanism involved in the action of naphthoquinones on trypanosomatid, possibly because of the generation of reactive oxygen species. These data merit further studies since plumbagin has been found to induce apoptosis in cancer cell lines by downregulating NF-κB, inducing caspase-3 activity and cell cycle arrest [62].

## 5. Conclusions

More than a century after the discovery of Chagas disease by Dr Carlos Chagas, this disease still represents a serious public health problem, and presents major challenges, including the requirement for a more efficient chemotherapy. Natural products represent a promising alternative for the treatment of several diseases, including protozoal infections. In this work, we investigated the antiparasitic effect of extracts of *P. auriculata* as well as its purified plumbagin. The crude root extract as well as the naphthoquinone were potent on different forms and strains of the parasite, despite having revealed a moderate profile of toxicity on mammalian cells. Ultrastructural analyses were carried out and the findings suggested that the exposure of trypomastigotes to Pb could result in autophagic death events. In addition, drug interactions between the reference drug and the root extract and plumbagin were also analyzed, revealing an additive effect for both combinations. Our set of results corroborates the literature data regarding the promising antiparasitic activity of naphthoquinones. These data corroborate the idea that natural products, especially isolated and purified compounds, deserve to be widely explored, as they provide important information and scaffolds for new drug candidates for the therapy of neglected tropical diseases.

## Figures and Tables

**Table 1:** Phenotypic activity (EC_50_ and EC_90_ μg/mL) of the *Plumbago auriculata* extracts (μg/mL) and of benznidazole (Bz) against intracellular forms (Tulahuen strain) and bloodstream trypomastigotes (BTs, Y strain) of *T. cruzi*, besides the cytotoxicity aspects upon the L929 cell line cultures and cardiac cells (CCs) (LC_50_, μg/mL), and their respective selectivity indexes (SIs).

	Intracellular Forms	L929	SI		BT		CC	SI
	96 h			2 h	24 h			
	EC_50_	LC_50_		EC_50_	EC_50_	EC_90_	LC_50_	
Bz	0.57 (0.3–0.8)	>100	>175	>100	2.96 (1.9–4.6)	5.9 (3.3–10.7)	>100	>34
Root	3.6 (2.8–4.6)	29.6 (24.0–36.5)	8.2	30.1 (16.9–53.4)	1.9 (1.2–2.8)	5.6 (2.5–12.5)	>100	>53
Branches	>100	>400	ND	>100	41.3 (24.7–68.8)	>100	>100	>2
Leaves	>100	>400	ND	>100	>100	>100	>100	ND
Flower	>100	>400	ND	>100	>100	>100	>100	ND

ND, not determined.

**Table 2:** Molecular properties of Lipinski’s rule of five using pkCSM tool.

Property	Reference	Bz	Plumbagin
Molecular Weight	≤500	260.253	188.18
LogP	≤5	1.1077	1.7175
Acceptors	≤5	5	3
Donors	≤10	1	1

**Table 3:** In silico ADMET analysis using pkCSM tool.

Property	Reference	Bz	Plumbagin
ABSORPTION			
Water solubility (log mol/L)	-	−2.723	−1.512
Caco2 permeability (log cm/s)	>0.9	0.655	1.215
Intestinal absorption (human, %)	<30% is poor	75.494	96.09
Skin permeability (log Kp)	>−2.5 is low	−2.735	−3.438
DISTRIBUTION			
P-glycoprotein substrate	No	Yes	No
P-glycoprotein I inhibitor	No	No	No
P-glycoprotein II inhibitor	No	No	No
VDss (human) (log L/kg)	Low is <−0.15, high is >0.45	0.234	−0.007
Fraction unbound (human)	-	0.325	0.58
BBB permeability (log BB)	Poor is <−1, high is >0.3	−0.863	−0.232
CNS permeability (log PS)	Penetrate is >−2, unable is <−3	−2.69	−2.911
METABOLISM			
CYP2D6 substrate	No	No	No
CYP3A4 substrate	-	No	No
CYP1A2 inhibitor	No	No	No
CYP2C19 inhibitor	No	No	No
CYP2C9 inhibitor	No	No	No
CYP2D6 inhibitor	No	No	No
CYP3A4 inhibitor	No	No	No
EXCRETION			
Total clearance (log ml/min/kg)	-	0.519	−0.032
TOXICITY			
AMES toxicity	No	Yes	No
Max. tolerated dose (human-log)	Low is ≤0.477, high is >0.477	1.029	0.705
hERG I inhibitor	No	No	No
hERG II inhibitor	No	No	No
Oral rat acute toxicity (LD_50_)	-	2.365	1.97
Oral rat chronic toxicity (log)	-	1.682	2.583
Hepatotoxicity	No	Yes	No
Skin Sensitization	No	No	No
*T. pyriformis* toxicity (log ug/L)	>−0.5 is toxic	0.285	−0.279
Minnow toxicity (log mM)	<−0.3 is toxic	2.06	1.271

**Table 4:** Phenotypic activity (EC_50_ and EC_90_ μM) of plumbagin (Pg) and of benznidazole (Bz) against intracellular forms (Tulahuen strain) and bloodstream trypomastigotes (BTs, Y strain) of *T. cruzi* and cytotoxicity (LC_50_, μM) upon L929 cultures and primary cardiac cell (CC), with their respective selectivity indexes (SIs).

	Intracellular Forms		L929	SI	BT				CC	SI
	96 h				2 h		24 h			
	EC_50_	EC_90_	LC_50_		EC_50_	EC_90_	EC_50_	EC_90_	LC_50_	
Bz	2.4 (1.4–3.4)	12 ( 6–18)	>200	>83	>50	>50	8.5 (8–9)	40.7 (39.5–41.9)	>200	>24
Pb	3.2 (2.2–4.2)	7.3 (3.3–11.3)	12 (8–16)	3	2.6 (2.5–2.7) *	6.15 (5.4–7.2) *	0.8 (0.2–1.4) *	1.7 (1.1–2.3) *	1.35 (0.9–1.7)	1.84

*, *p* < 0.05 determined via ANOVA.

## Data Availability

All relevant data are included in the manuscript.

## Supplementary Materials

The following supporting information can be downloaded at: https://www.mdpi.com/article/10.3390/pharmaceutics15051535/s1: Figure S1: Plumbagin structure; Figure S2: GC-MS for Plumbagin; Figure S3: GC-MS for Plumbagin (CG-EM m/z 188 [M]+ (100), 131 (41), 173 [M-CH3] (25), 132 (24), 160 (21)); Figure S4: ^1^H NMR spectrum for Plumbagin; Figure S5: ^13^C NMR spectrum for Plumbagin; Figure S6: COSY ^1^H x ^1^H spectrum for Plumbagin; Figure S7: ^13^C DEPT 135 NMR spectrum for Plumbagin; Figure S8: HSQC ^1^H x ^13^C spectrum for Plumbagin; Figure S9: ^1^H x ^13^C-HMBC spectrum for Plumbagin; Table S1: NMR data of the isolated plumbagin.

## Abbreviations

ADMET	Absorption, distribution, metabolism, excretion and toxicity
BTs	Bloodstream trypomastigotes
Bz	Benznidazole
CCs	Cultures of cardiac cells
EC_50_	Compound concentration that reduces 50% of the number of parasites
EC_90_	Compound concentration that reduces 90% of the number of parasites
CD	Chagas disease
CDCl_3_	Deuterated chloroform
CEUA	Committee of Ethics for the Use of Animals
COSY	Homonuclear correlation spectroscopy
DEPT	Distortionless enhancement by polarization transfer
DMSO	Dimethyl sulfoxide
DTUs	Discrete typing units
FBS	Fetal bovine serum
FDA	Food and Drug Administration
FICI	Fractional inhibitory concentrations index
hERG	Human ether-à-go-go-related gene
HMBC	Heteronuclear multiple bond correlation
HSQC	Heteronuclear single quantum coherence
LC_50_	The concentration that kills 50% of the parasite
Nf	Nifurtimox
NMR	Nuclear magnetic resonance
OsO4	Osmium tetroxide
Pb	Plumbagin
P-gp	P-glycoprotein
pKCSM	Small-molecule pharmacokinetic and toxicity properties
SEM	Scanning electron microscopy
SIs	Selectivity indexes
*T. cruzi*	*Trypanosoma cruzi*
TEM	Transmission electron microscopy
TMS	Tetramethylsilane
ΣFICIs	Sum of fractional inhibitory concentration indexes
µg/mL	Micrograms per milliliter
μM	Micromolar
