# Supplementary material for: Antiparasitic Activity of Plumbago auriculata Extracts and Its Naphthoquinone Plumbagin against Trypanosoma cruzi
Source: Pharmaceutics. 2023 May 19;15(5):1535. doi: 10.3390/pharmaceutics15051535 (PMC10221501; doi:10.3390/pharmaceutics15051535)
Supplement: Supplementary file 1 [file pharmaceutics-15-01535-s001.zip › pharmaceutics-2372714-supplementary.pdf]

**Supplementary methods for:** (Suppl S1)

## Plumbagin structure

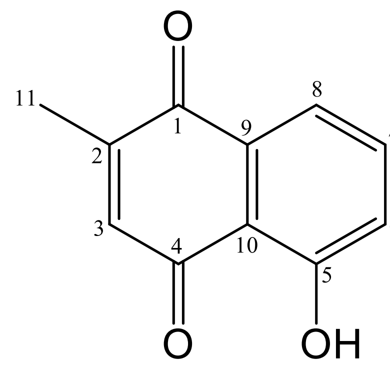


**Figure S1:** Plumbagin structure

## NMR Spectroscopy

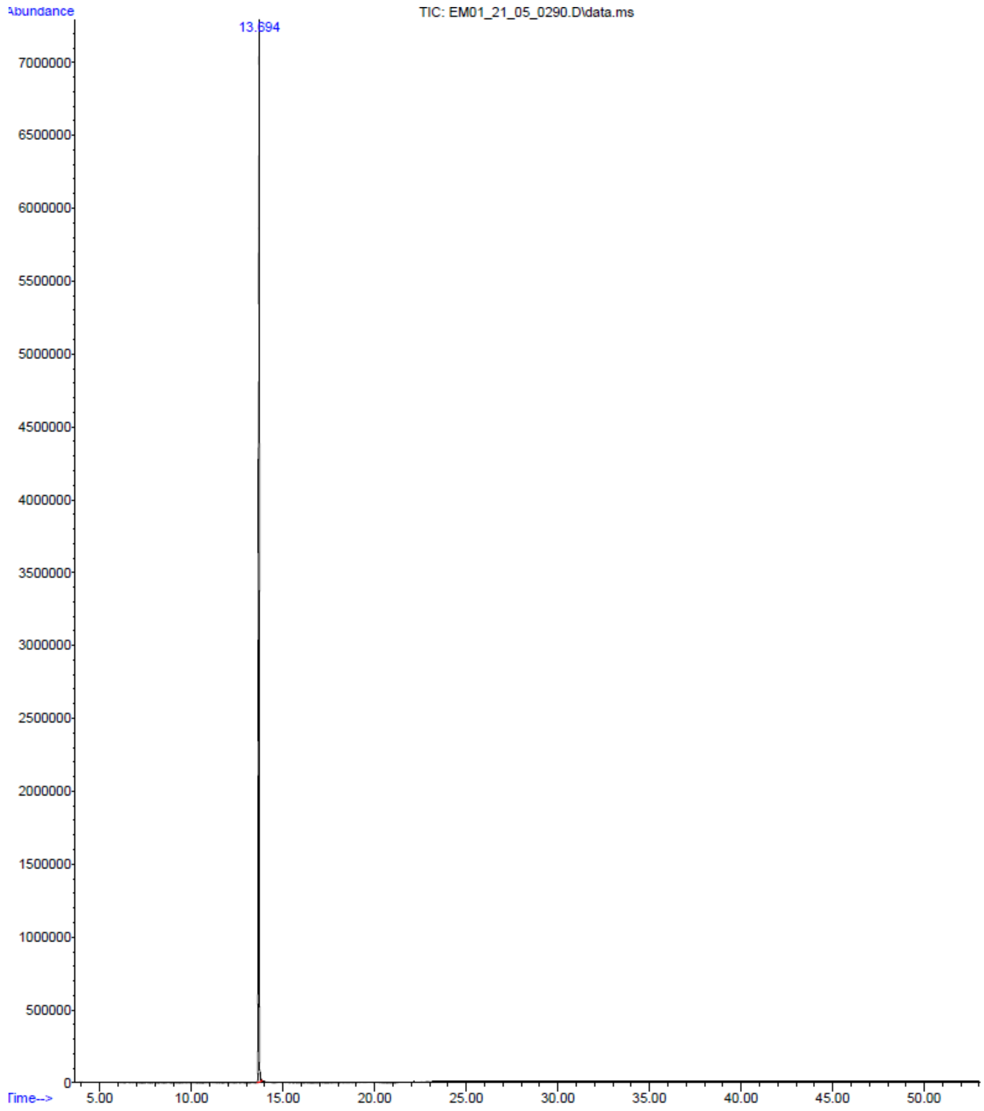


**Figure S2:** GC-MS for Plumbagin

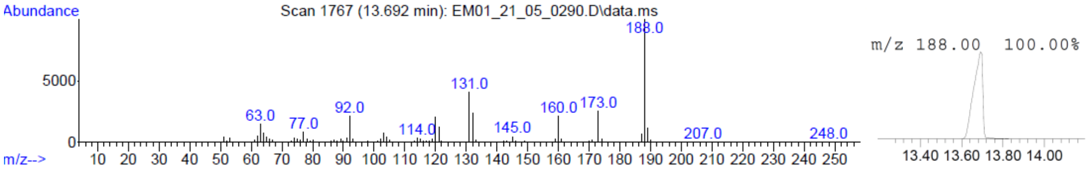


**Figure S3:** GC-MS for Plumbagin (CG-EM *m/z* 188 [M]+ (100), 131 (41), 173 [M - $CH_3$] (25), 132 (24), 160 (21)).

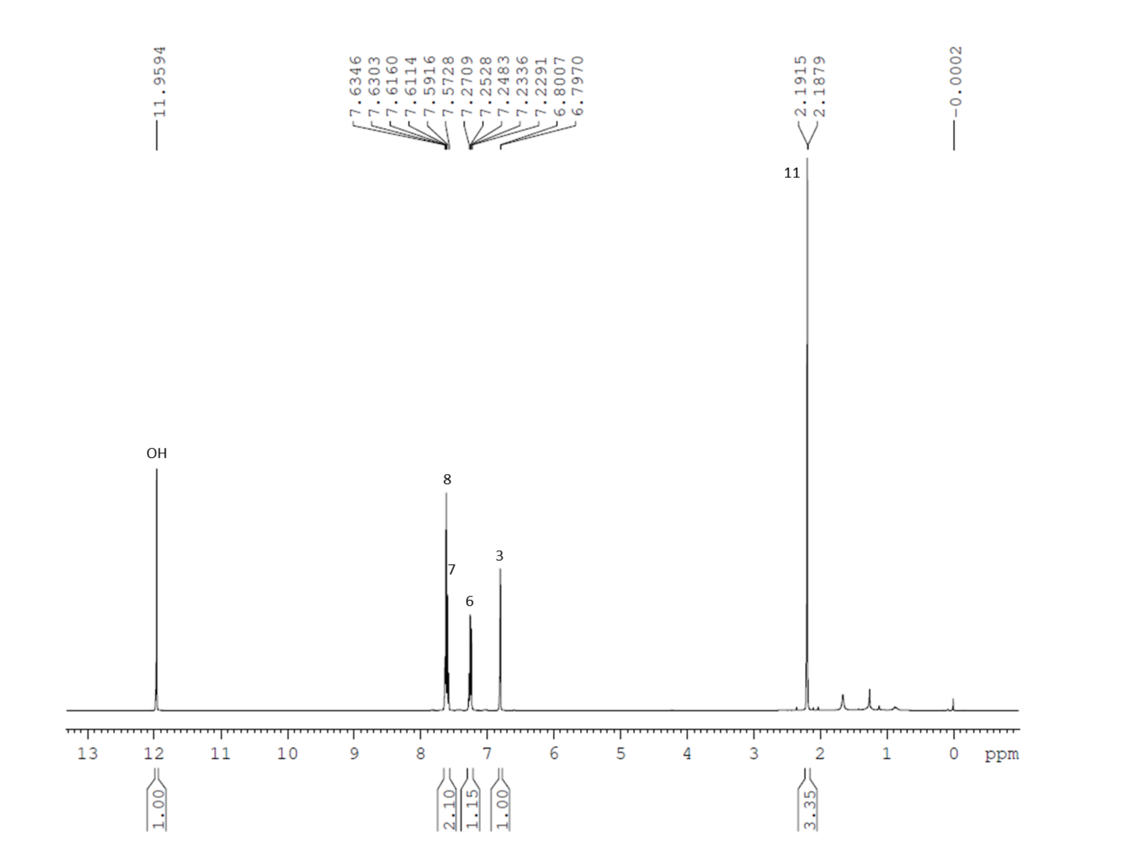


**Figure S4:** $^{1}H$ NMR spectrum for Plumbagin

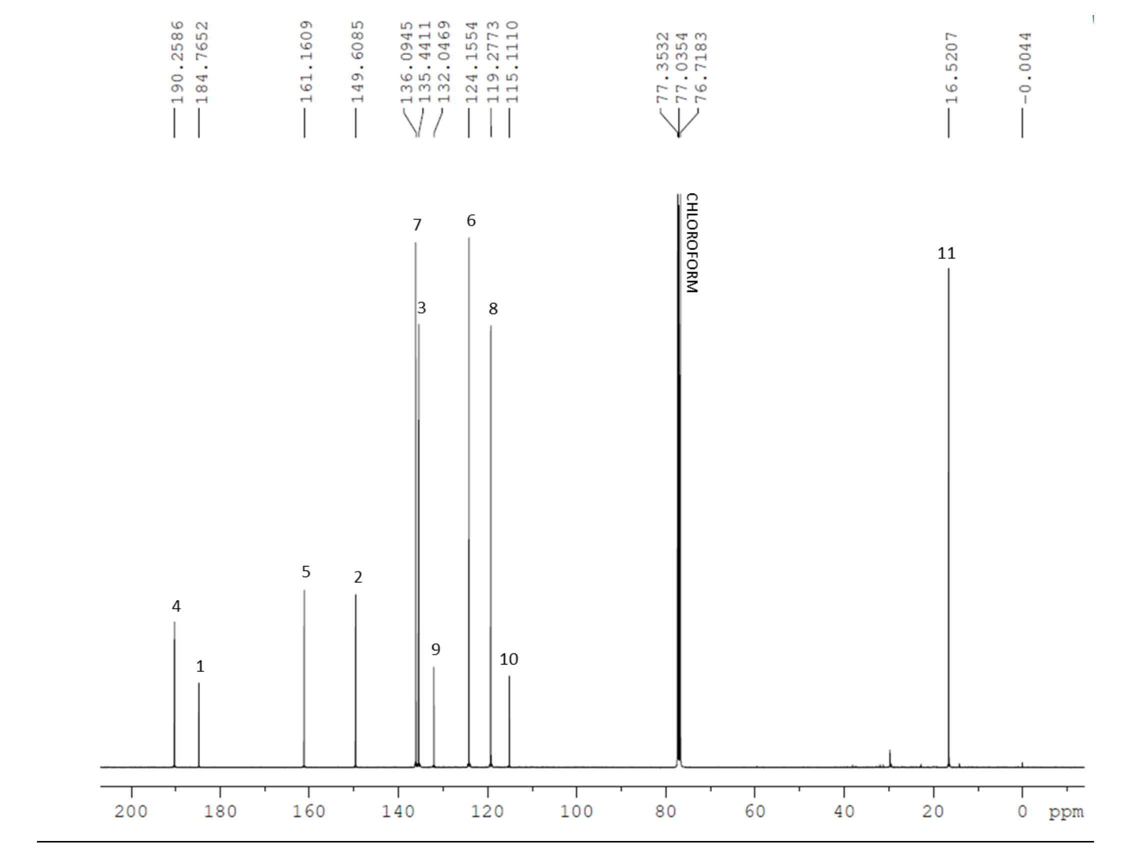


**Figure S5:** $^{13}C$ NMR spectrum for Plumbagin

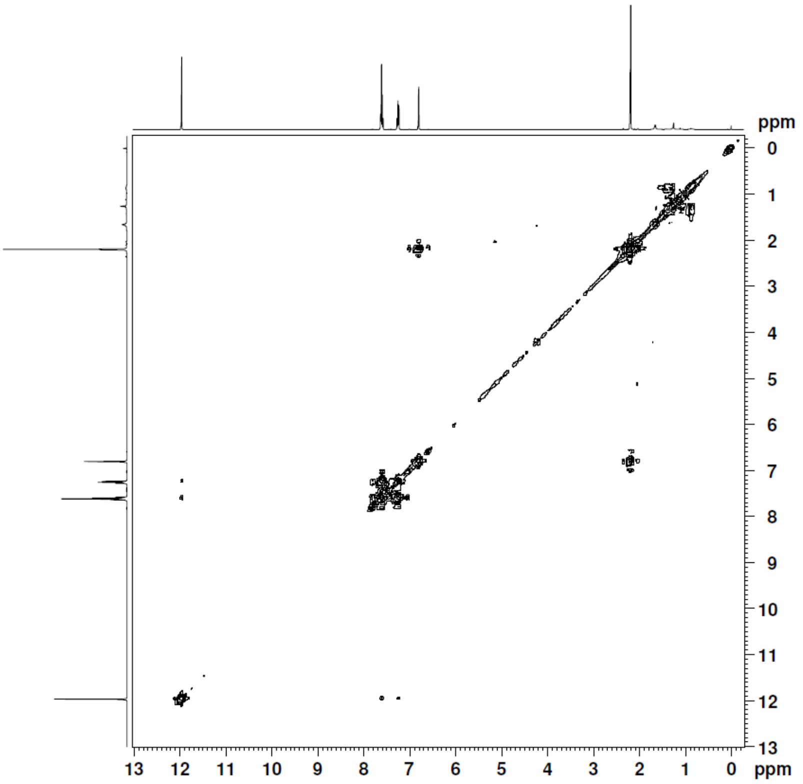


**Figure S6:** COSY $^1H$ x $^1H$ spectrum for Plumbagin

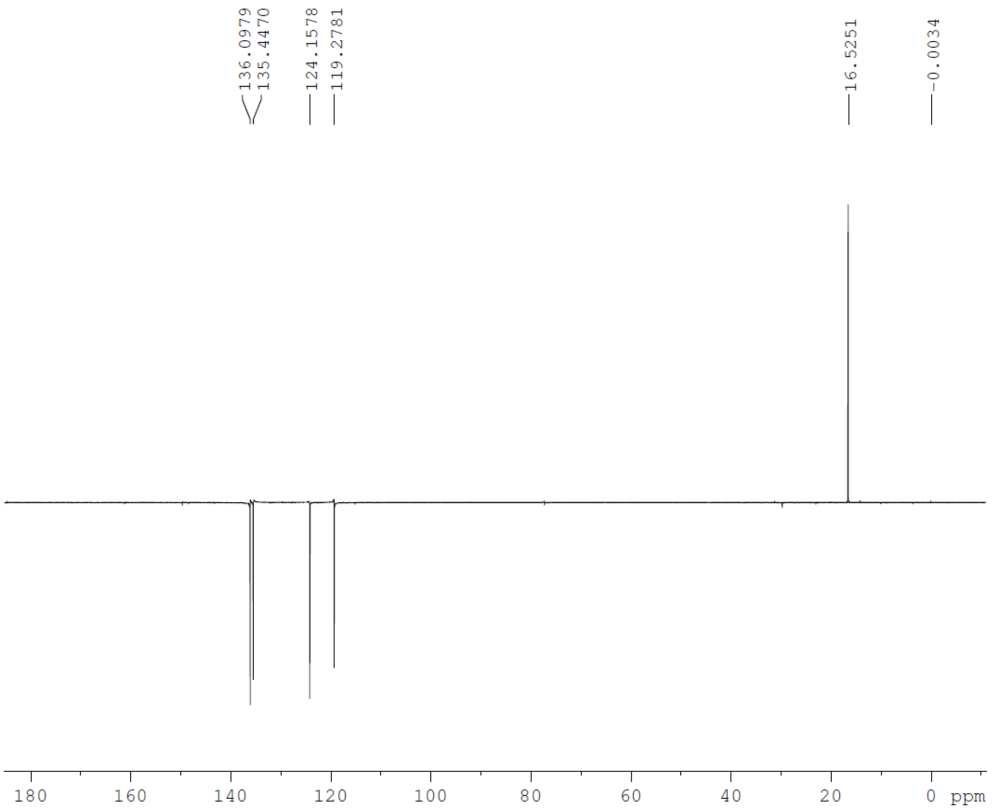


**Figure S7:** $^{13}C$ DEPT-135 NMR spectrum for Plumbagin

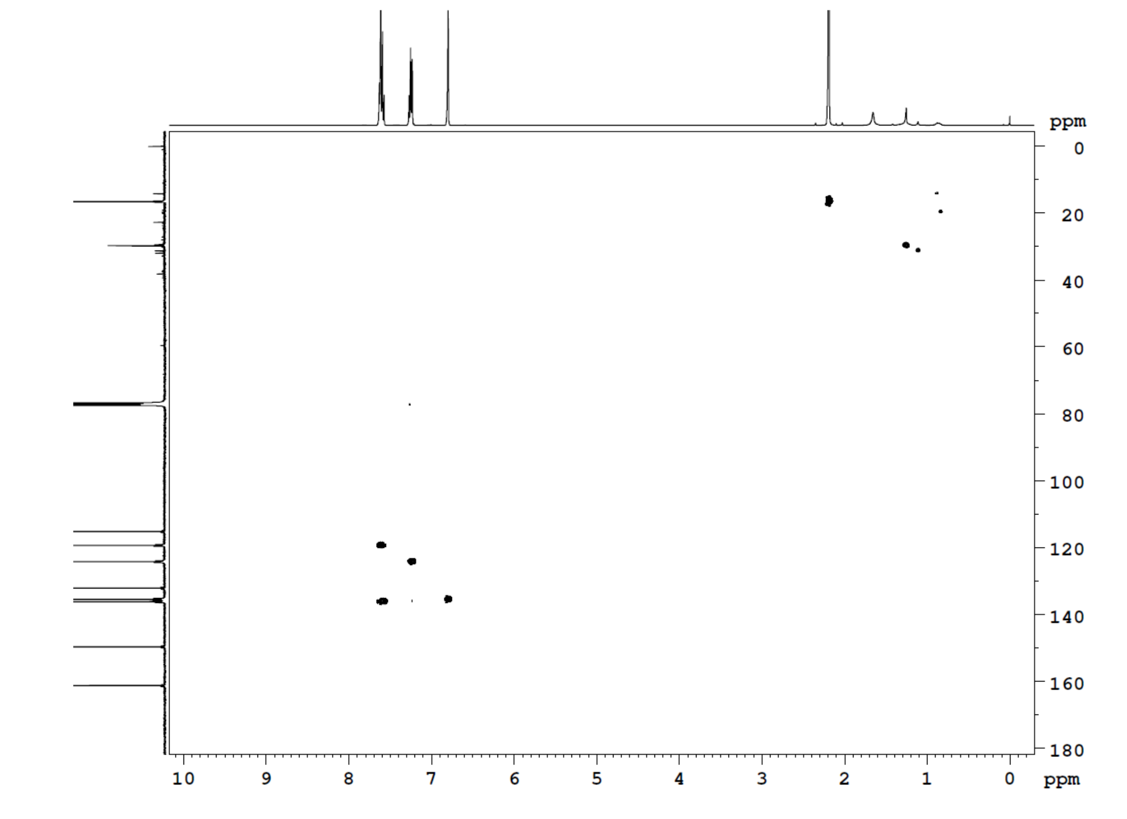


**Figure S8:** HSQC $^1H$ x $^{13}C$ spectrum for Plumbagin

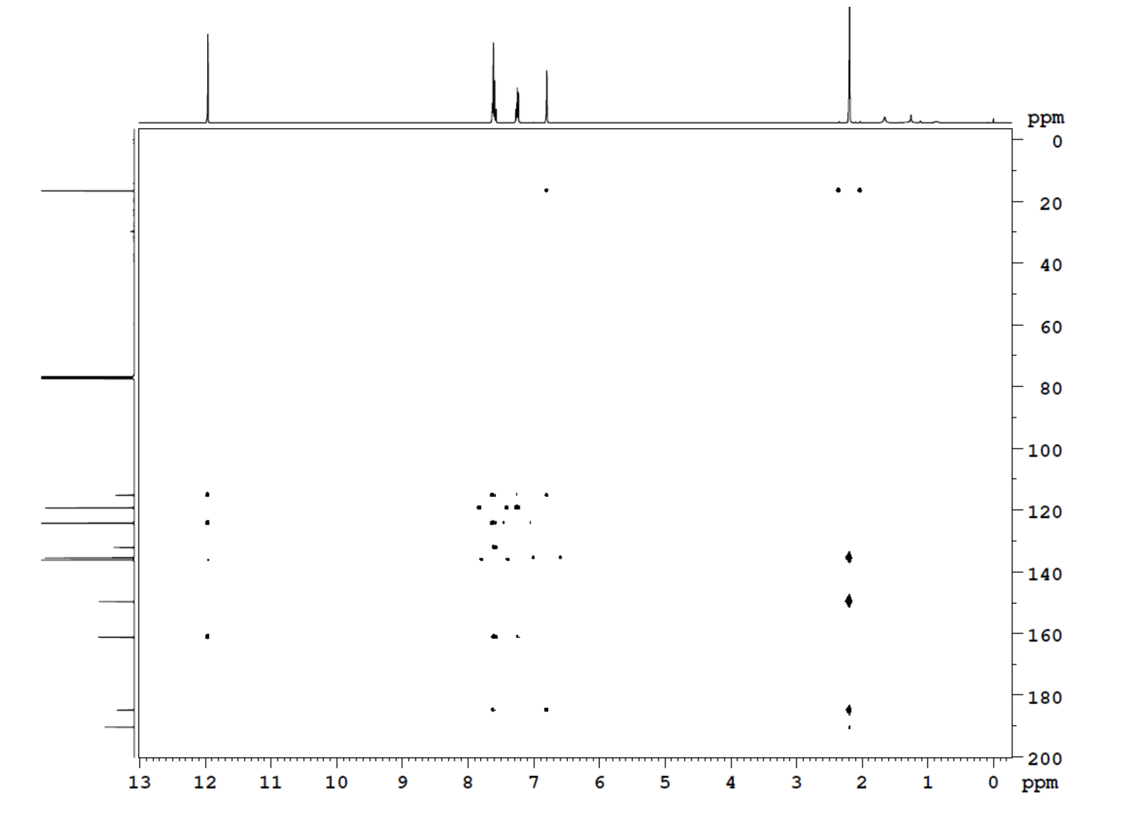


**Figure S9:** $^1H$ x $^{13}C$-HMBC spectrum for Plumbagin

**Table S1:** NMR data of the isolated plumbagin

| Position | The isolated plumbagin[a] | | The reported plumbagin[a] | | The reported plumbagin[a] | |
|---|---|---|---|---|---|---|
| | $^{1}$H NMR | $^{13}$C NMR | $^{1}$H NMR | $^{13}$C NMR | $^{1}$H NMR | $^{13}$C NMR |
| 1 | | 184.4 | | 184.7 | | 184.7 |
| 2 | | 149.6 | | 149.5 | | 149.6 |
| 3 | 6.80 (*d*, H3; *J*=1.48) | 135.4 | 6.8 (*d*, H3) | 135.4 | 6.7 (*s*, H-3) | 136.0 |
| 4 | | 190.2 | | 190.2 | | 190.2 |
| 5 | | 161.1 | | 161.1 | | 161.1 |
| 6 | 7.24 (*dd*, H6; $J_1$=7.68, $J_2$=1.80) | 124.1 | 7.23 (*m*, H6) | 124.1 | 7.22 – 7.20 (*m*, H-6) | 124.1 |
| 7 | 7,59 (*t*, H7; *J*=7.52) | 136.0 | 7.60 (*m*, H7) | 136.0 | 7.59 (*m*, H7) | 135.4 |
| 8 | 7.62 (*dd*, H8; $J_1$=7.56, $J_2$=1.72) | 119.2 | 7.60 (*m*, H8) | 119.0 | 7.55 (*m*, H8) | 119.2 |
| 9 | | 132.0 | | 132.0 | | 132.0 |
| 10 | | 115.1 | | 115.0 | | 115.2 |
| 11 | 2.19 (*d*, $CH_3$; *J*= 1.44) | 16.5 | 2.18 (*d*, H11) | 16.5 | 2.16 (*s*, -$CH_3$) | 16.1 |
| -OH | 11.96 (*s*, OH) | | 11.95 | | 11.94 (*brs*, -OH) | |

[a] Measured in $CDCl_3$
